# Vitamin C-induced hyperoxaluria causing reversible tubulointerstitial nephritis and chronic renal failure: a case report

**DOI:** 10.1186/1752-1947-1-155

**Published:** 2007-11-27

**Authors:** Shradha Rathi, William Kern, Kai Lau

**Affiliations:** 1 Department of Medicine, The University of Oklahoma Health Sciences Center, 1100 N. Lindsay, Oklahoma City, OK 73104, USA; 2 Department of Pathology, The University of Oklahoma Health Sciences Center, 1100 N. Lindsay, Oklahoma City, OK 73104, USA; 3 Department of Medicine, The University of Oklahoma Health Sciences Center, 1100 N. Lindsay, Oklahoma City, OK 73104, USA

## Abstract

**Abstract:**

Vitamin C is a precursor of oxalate and promoter of its absorption, potentially causing hyperoxaluria. Malabsorption causes Calcium (Ca) chelation with fatty acids, producing enteric hyperoxaluria.

**Case:**

A 73-year-old man with both risk factors was hospitalized with serum creatinine of 8.4 mg/dL (versus 1.2 mg/dL four months earlier) (normal 0.6–1.3 mg/dL). Given his oxalate-rich diet, chronic diarrhea, and daily 680 mg vitamin C and furosemide, we postulated Ca oxalate-induced nephropathy, a diagnosis confirmed by documenting hyperoxaluria, and finding of diffuse intraluminal crystals and extensive interstitial fibrosis on biopsy. He was hemodialysed 6 times to remove excess oxalate. Two weeks off vitamin C, his creatinine spontaneously fell to 3.1 mg/dL. Three months later, on low oxalate diet and 100 mg vitamin B6, urine oxalate to creatinine ratio decreased from 0.084 to 0.02 (normal < 0.035), while creatinine fell and stayed at 1.8 mg/dL.

**Conclusion:**

1) High-dose vitamin C can induce hyperoxaluric nephropathy and progressive renal failure, especially if aggravated by diarrhea, oxalate-rich diet, metabolic acidosis, and dehydration. 2) The diagnosis should be suspected in unexplained renal insufficiency when associated with these risk factors. 3) Since prompt treatment could avert end-stage renal disease, we recommend monitoring urinary oxalate in patients on high-dose vitamin C and renal biopsy if necessary.

## Introduction

In humans, oxalate is an end product of metabolism. Absorbed from gut and produced endogenously, it must be excreted to prevent systemic oxalosis. Due to low solubility of Ca oxalate (CaOx), primary hyperoxaluria, caused by enzymatic deficiency, produces nephrolithiasis, nephrocalcinosis, and progressive renal failure. Less recognized is subacute insidious nephropathy from secondary causes like excessive vitamin C and malabsorption. Vitamin C is a precursor of oxalate, liable to produce hyperoxaluria [1-5]. It can also increase oxalate absorption, further accentuating the hyperoxaluria. In malabsorption, Ca chelates with fatty acids, generating enteric hyperoxaluria. Chronically, these risk factors predispose to nephrolithiasis and progressive renal failure, similar to primary hyperoxaluria [6,7]. We here report a patient with severe renal failure due to these acquired risk factors, plus dehydration and hypocitraturia from diarrhea-induced metabolic acidosis. He responded well to stopping vitamin C and low oxalate diet, regaining enough function to avoid chronic dialysis.

## Case Presentation

**Figure 1:**
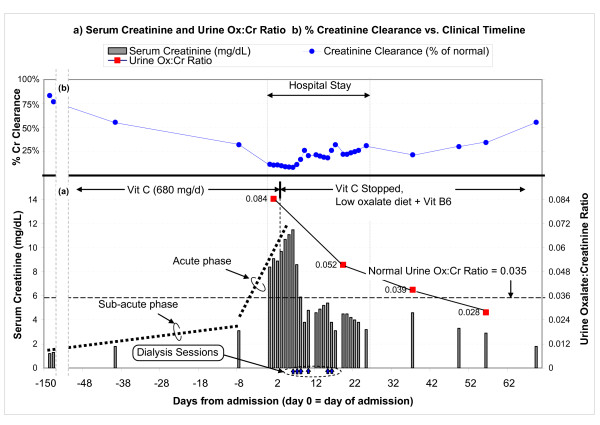
**Serum creatinine, urine oxalate:creatinine ratio, and creatinine clearance vs. clinical timeline**. (a) The chart shows the trend of serum creatinine (gray bars, with the values shown on the left axis), starting from a baseline of 1.2 mg/dL just over 4 months ago, gradually increasing up to 3.1 mg/dL 8 days ago, and rapidly increasing to 8.4 mg/dL on admission (day 0). The urine oxalate:creatinine ratio (red squares connected by lines, with values shown on the right axis) clearly shows hyperoxaluria at admission (0.084 at day 1, compared to a normal of 0.035). Vitamin C was stopped on day 4, and creatinine started improving after 2 days. (b) Renal function in terms of creatinine clearance (% of normal) is also shown.

A 73-year-old man was hospitalized for chronic diarrhea and serum creatinine of 8.4 mg/dL (vs. 1.2, 1.8, and 3.1, respectively, 4 months, 5 weeks, and 8 days earlier) (Fig 1a). He had a past history of chronic alcoholism, atrial fibrillation, hypertension, heart failure, and hypothyroidism (all resolved or controlled).

On examination, he appeared chronically ill, afebrile, alert, and fully oriented. Blood pressure was 121/75 mmHg. O_2 _saturation was normal. He weighed 68 Kg, 2 Kg lighter than 3 months ago. His lungs were clear and heart rhythm was sinus. His abdomen and extremities were normal, without edema or asterixis.

Admission blood tests were remarkable for creatinine of 8.4 mg/dL, which quickly became 11 mg/dL despite fluids (Fig 1a). HCO_3 _was 19 mM (22–29 mM). Anion gap was 14. Ca was 10.4 mg/dL (normal 8.5–10.5 mg/dL). Ionized Ca was high at 1.24 mM. Phosphorus was 4 mg/dL (normal 2.5–8.5 mg/dL). CBC was abnormal for hemoglobin of 9.2 g/dL and persistent megaloblastosis. Urine pH was 5. Specific gravity was 1.020, without casts or eosinophils. Urine protein:creatinine ratio was 1.2. Kidneys were 10.1 × 7.4 × 5.1 cm (right) and 10.6 × 3.8 × 4 cm (left) on ultrasound.

Serial creatinine (Fig 1a) suggested chronic slowly-progressive failure, until 5 weeks ago, when creatinine rose abruptly. Since there was no history or evidence for diabetes, accelerated hypertension, nephritis, contrast dye, sepsis, allergic reactions, obstruction, thrombo-emboli, or volume overload or depletion, we postulated nephrotoxins exposure at home to explain his slow, insidious relentless renal impairment. Diet scrutiny revealed excessive beans and chocolate, rich in oxalate. Medication review showed daily 5 mg lisinopril, 75 μg levothyroxine, 800 mg MgO, 650 mg CaCO3, 500 mg niacin, 10,000 IU vitamin A, 500 mg vitamin C, and a multivitamin with 180 mg of vitamin C.

Since urinalysis was negative and proteinuria was minimal, oxalate-induced tubulointerstitial nephritis was proposed, given history of alcoholism, chronic diarrhea, and high-dose vitamin C. On day 2, 24-hour urine was submitted for oxalate. Given his weight loss, renal failure, and hypercalcemia, multiple myeloma was considered unlikely given negative serum and urine protein electrophoresis and negative bone marrow exam. Other causes for hypercalcemia were excluded by normal PTH, PTH-related peptide, 1,25 (OH)_2 _vit D, 25 OH vit D, alkaline phosphatase, and free T4. Neoplasms were excluded by CT scans of chest, abdomen, and pelvis, and endoscopies.

His creatinine continued to rise despite drinking plenty of fluids and stopping lasix and lisinopril (Fig 1a). Salt depletion was excluded by lack of fall in creatinine despite several liters of saline, arguing against dehydration as the cause for his acute renal failure. While still waiting for urine oxalate results, which were delayed due to erroneous submission of un-acidified aliquot, we stopped vitamin C on day 4. Hypercalcemia was attributed to CaCO_3_, vitamins A and D, as it resolved off these agents. Diarrhea was attributed to MgO, vitamin A, and niacin, all known to have cathartic potential. Indeed, diarrhea resolved once they were stopped. These responses reinforced the suspicion of nephrotoxic "over-the-counter" medications.

He received 6 hemodialysis treatments to correct metabolic acidosis (that otherwise reduces urine citrate and predisposes to Ca precipitation [8] and to remove excess oxalate (from presumed enhanced absorption and impaired excretion). Discontinuation of vitamin C resulted in slow steady recovery of renal function. Within days, serum creatinine started falling and continued spontaneously (Fig 1a). Nine days after his last dialysis, creatinine was 3.1 mg/dL. Six weeks later, while on metolazone (to reduce urine Ca), a low oxalate diet, and 100 mg daily vitamin B6, his creatinine was 1.8 mg/dL. It stayed there 3 months after presentation. Urine oxalate to creatinine ratio steadily decreased, from 0.084 pre-treatment to 0.02 (normal < 0.035) (Fig 1a).

**Figure 2:**
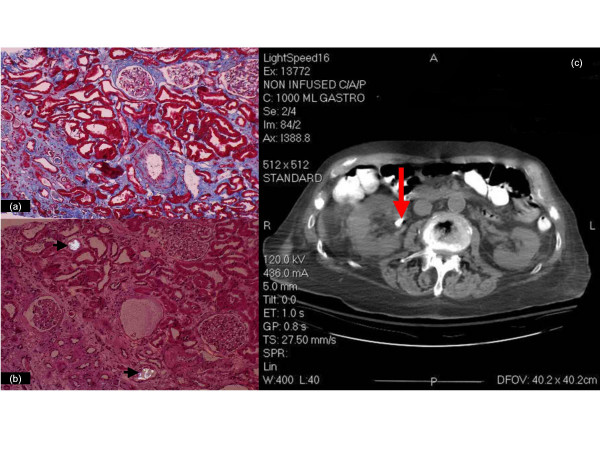
**Slides from left kidney biopsy and CT image of abdomen**. There is extensive interstitial fibrosis and tubular atrophy, with marked medial fibrosis in an artery on the lower right (trichrome/10x) (a); bright crystals are seen under partial polarization within the lumens of two tubules (arrows) (H&E/20x) (b). A 10 mm calculus is seen in the right extra renal pelvis (arrow) in the CT (c).

The presumptive diagnosis of oxalate-induced interstitial nephritis was confirmed on day 14 by a renal biopsy, showing extensive interstitial fibrosis, tubular atrophy (Fig 2a), and diffuse intraluminal birefringent crystals under polarized light (Fig 2b). CAT scan documented scattered renal calcifications and a 1-cm calculus (Fig 2c). Urine from day 2 showed increased oxalate:creatinine ratio excretion of 0.084 (Fig 1a), biochemically corroborating the clinical diagnosis and biopsy findings.

The hyperoxaluria was attributable to vitamin C, as it resolved on discontinuation of vitamin C (urine oxalate:creatinine ratio 0.052, Fig 1a). A low oxalate diet further reduced this ratio to 0.039 and to 0.028 (Fig 1a). We did not measure urine citrate during his peak renal failure since severe metabolic acidosis is expected to non-specifically suppress excretion. However, 11 and 30 days post-discharge, when serum HCO_3 _approached normal (22 and 21 mM) and creatinine substantially improved (4.6 and 2.9 mg/dL), we still documented markedly reduced urine citrate (respectively, 14 mg and 18 mg per gram of creatinine; normal being ≥ 250). These results suggest significant ongoing metabolic acidosis preceding or paralleling the steady rise in serum creatinine in previous months.

## Discussion

Urinary oxalate is derived from endogenous production and absorption from exogenous sources. Hyperoxaluria (urine oxalate above the normal range of 10–35 mg/24 hr) can be primary or secondary. Primary hyperoxaluria results from genetic defects in glyoxylate metabolism, producing nephrolithiasis, nephrocalcinosis, and progressive renal insufficiency [9]. Secondary hyperoxaluria is acquired from enteric causes or ethyl glycol intoxication. Normally, Ca binds most of the intestinal oxalate, with subsequent stool CaOx elimination. Accordingly, only 4 to 12% of enteric oxalate is normally absorbed. After small bowel bypass, Ca complexes with poorly absorbed fatty acids, leaving behind excess unbound oxalate for absorption, 65 to 80% of which occurs in the colon [6,7]. This mechanism also explains the enteric hyperoxaluria in chronic diarrhea and malabsorption.

Another important but under-appreciated etiology of secondary hyperoxaluria is increased synthesis from vitamin C [1-5]. We previously reported the quantitative dose-response relationship in a patient receiving vitamin C solely from parenteral nutrition [5]. If coexisting, malabsorption and high-dose vitamin C could potentiate the hyperoxaluria induced by each other.

Clinically, hyperoxaluria presents in one of several ways, depending on the severity, chronicity, etiologies and co-factors. First, at one extreme, as in ethylene glycol intoxication, fulminant acute renal failure from intraluminal obstruction can develop, due to excessive oxalate production and profound hyperoxaluria. Second, at the other end, intermittent hyperoxaluria could cause episodic painful renal colic from small punctuate Ca Ox calculi. Despite short-term morbidities, few suffer from serious long-term renal insufficiency. The third mode of clinical presentation is illustrated by primary hyperoxaluria.

Our patient demonstrates the fourth mode of manifestation in that insidious renal failure, easily missed, slowly evolves over weeks to months, unbeknown to patients and physicians until renal function is compromised by >50–70 %. Typically, for diffuse intraluminal crystal deposits and extensive interstitial fibrosis to develop, the course is protracted. While the first three modes (ethylene glycol intoxication, episodic stone attacks, and primary hyperoxaluria) are clinically symptomatic and promptly treated, the 4^th ^mode is generally quiescent, asymptomatic, undiagnosed, and untreated for months, like our patient, unless tests reveal incidental unexpected progressive renal failure.

**Figure 3:**
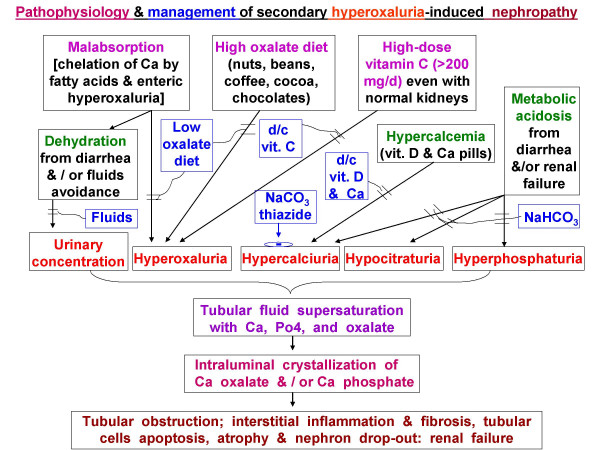
Pathophysiology and management of hyperoxaluric nephropathy.

In our patient, intratubular luminal precipitation of Ca oxalate was promoted by four pathogenic factors (Fig 3): (1) high urine specific gravity (due to diarrhea-induced dehydration), (2) hyperoxaluria from all three potentiating mechanisms (oxalate-rich diet, 680 mg daily vitamin C, and possible malabsorption), (3) relative hypercalciuria (due to furosemide, chronic metabolic acidosis, and hypercalcemia, caused by CaCO3 pills and vitamin A and D), and (4) hypocitraturia (due to metabolic acidosis, initially from chronic diarrhea and later aggravated by progressive renal failure).

The pathophysiology of hyperoxaluria-induced nephropathy is schematized in Fig 3. Tubular fluid supersaturated with Ca oxalate crystals causes luminal obstruction, macrophage recruitment, up-regulated reactive oxygen species, pro-inflammatory & pro-fibrogenic cytokines, creating a vicious cycle of interstitial nephritis, relentless injuries, apoptosis, necrosis, fibrosis, with eventual nephron drop-out and tubular atrophy. Initially, hypocitraturia from stool alkali losses is modest, but with worsening renal failure, metabolic acidosis and hypocitraturia become more severe. Nephron losses further elevate blood and filtered oxalate, raising tubular fluid oxalate concentration in surviving nephrons. These changes exacerbate the risks for luminal crystals precipitation, tubular obstruction, inflammation, and interstitial nephritis. As more tubules die out, the vicious cycle is exaggerated. This could explain the steep increase in serum creatinine from 1.8 to 8.4 mg/dL (representing 44 % loss in creatinine clearance in 5 weeks before admission) vs. the gentle rise from 1.2 to 1.8 mg/dL (representing 28% loss in clearance over the preceding 4 months) (Fig 1b).

Treatment of hyperoxaluria-induced nephropathy depends on interrupting the pathophysiology (Fig 3), specifically, by increased fluids, low oxalate diet, discontinuing vitamins A, C, D, and CaCO_3_, prescribing thiazide (or metolazone if creatinine is elevated, to reduce urine Ca), and NaHCO_3 _to increase urine citrate. Metabolic acidosis is known to markedly decrease tubular fluid citrate, the predominant endogenous inhibitor of Ca oxalate precipitation [8]. In patients with malabsorption, underlying etiologies should be addressed, and Na or K alkali salts (citrate or bicarbonate) should minimize metabolic acidosis, increase urine citrate, and reduce urine Ca. (Fig 3). We counseled continued alcohol abstinence in our patient. All identifiable causes of hypercalcemia and hypercalciuria were eliminated, and we empirically added vitamin B6, which promotes conversion of glyoxylate to glycine instead of oxalate.

Unfortunately, vitamin C-induced hyperoxaluria is often missed or diagnosed late in the course of renal failure, and for several reasons. One, increasingly individuals take daily multi-vitamins or high-dose vitamin C on their own. Second, current commercial preparations contain vitamin C typically several-fold over the adult daily requirement of 60 mg. Most juices, soft drinks, and diet supplements contain > 120 mg of vitamin C per 8 oz. These add greatly to food-derived oxalate. Third, self-prescribed medications are not routinely checked or discovered by physicians, as the potential adverse hyperoxaluric consequence is generally unrecognized. Fourth, the causal relationship between hyperoxaluria and renal failure is also under-appreciated. Finally, when advanced renal insufficiency emerges, the focus is shifted to uremia management. Identifying and defining the etiology is seldom exhaustively undertaken or usually unsuccessful.

## Conclusion

1) High-dose vitamin C can induce hyperoxaluric nephropathy and progressive renal failure, especially with diarrhea, oxalate-rich diet, metabolic acidosis, and dehydration. 2) The diagnosis should be suspected in unexplained, slowly evolving renal insufficiency, particularly if additional risk factors coexist. 3) Since prompt treatment could prevent end-stage renal disease, we recommend a high index of suspicion, careful review of diets and all medications, closely monitoring renal function and oxalate excretion in patients on vitamin C.

## Competing interests

The author(s) declare that they have no competing interests.

## Authors' contributions

SR did the initial workup and follow-up. KL made the diagnosis and treated the patient. Both SR and KL plotted the graphs and drafted the manuscript. WK was responsible for interpretation of biopsy findings. All authors read and approved the final manuscript.

## Consent

Written consent for the publication was obtained from patient's wife as the patient died of a cardiac event 5 months after presentation.
